# Pelvic floor rehabilitation in the treatment of mixed urinary incontinence among women

**DOI:** 10.1186/s43166-021-00087-w

**Published:** 2021-12-02

**Authors:** Nehad Mohamed Elshatby, Mohamed Hassan Imam, Mohamed Shafik Shoukry, Marwa Mohamed Hassan, Emmanuel Kamal Aziz Saba

**Affiliations:** 1 Present address: Faculty of Medicine, Department of Physical Medicine, Rheumatology and Rehabilitation, Alexandria University, Alexandria, Egypt; 2 Department of Genitourinary Surgery, Alexandria University, Alexandria, Egypt

**Keywords:** Mixed urinary incontinence, Biofeedback, Neuromodulation, Pelvic floor muscle training, Rehabilitation

## Abstract

**Background:**

Mixed urinary incontinence (MUI) is a common underreported problem among females; it has a major effect on patients’ quality of life. Treatment may be difficult since a single modality cannot be enough to alleviate both the urge and the stress symptoms. Biofeedback-assisted pelvic floor muscle training (PFMT) has a great role in strengthening the pelvic floor muscles especially when accompanied by electrical stimulation. Neuromodulation is another safe well-tolerated method that may improve symptoms of female voiding dysfunction. There are no previous studies that assessed the efficacy of biofeedback-assisted pelvic floor muscle training versus two different types of peripheral neuromodulation which are transcutaneous posterior tibial nerve stimulation (TPTNS) and anogenital neuromodulation in the treatment of mixed urinary incontinence among women. The aim of this work is to study the effectiveness of biofeedback-assisted pelvic floor muscle training with electrostimulation versus two different methods of peripheral neuromodulation techniques in the treatment of women with MUI. Patients were subjected to history taking, assessment questionnaires (Questionnaire for female Urinary Incontinence Diagnosis (QUID), Australian Pelvic Floor Questionnaire (PFQ), and International Consultation on Incontinence Questionnaire-Urinary Incontinence-Short Form (ICIQ-UI-SF)), clinical examination, and manometric pressure assessment. The patients were allocated randomly into three groups. Group I received biofeedback-assisted pelvic floor muscle training and faradic electrical stimulation, group II received posterior tibial neuromodulation, and group III received anogenital neuromodulation.

**Results:**

The present study included 68 non-virgin female patients with mixed urinary incontinence. Significant improvement was noticed in the three studied groups on the subjective and objective levels. No statistically significant difference was reported between the studied groups following the different types of intervention.

**Conclusions:**

Biofeedback-assisted pelvic floor muscle training with electrostimulation is as effective as anogenital neuromodulation and posterior tibial neuromodulation in the treatment of mixed urinary incontinence among females.

**Trial registration:**

PACTR, PACTR202107816829078. Registered 29 July 2021 - Retrospectively registered.

## Background

Mixed urinary incontinence (MUI) is a subtype affecting one in every three women suffering from urinary incontinence. It is characterized by a combination of stress and urge symptoms in the same patient. It has a major effect on a woman’s quality of life (QOL) [1, 2].

The Questionnaire for female Urinary Incontinence Diagnosis (QUID) is a valuable research instrument, as history is often the most important contributor to diagnosis [3]. Women with MUI have more severe symptoms and do not respond well to treatment than others with only one type of UI. Conservative treatment is the first line of management [1].

Pelvic floor muscle training (PFMT) is an efficient technique to improve symptoms of all types of UI by strengthening PFM and increasing their endurance and power [4]. It is highly recommended by the European Association of Urology (EAU) 2018 updated guidelines on UI [5]. Biofeedback (BF) is commonly linked to pelvic floor muscle training (PFMT) for women with urinary incontinence. It teaches patients how to voluntarily contract the pelvic floor muscles efficiently to prevent leakage [6]. Biofeedback apparently adds the benefit of psychological support to the patient who becomes in direct contact with the health professional, hence involved actively in the rehabilitation program, thus gaining great therapeutic effect [6, 7].

The surged faradic current is used to enforce the effect of active exercise by improving the strength and vascularity of various groups of muscles including pelvic floor muscles [8].

Neuromodulation is the modulation of the physiologic behavior of the nerve by electrical stimulation. Most of the recent studies revealed that neuromodulation inhibits urge sensation without influence on the urethral resistance or detrusor muscle contraction [9]. Braun and colleagues found that neuromodulation affects the supraspinal centers in humans, as they recorded reproducible cortical potentials in the electroencephalograms of patients [9]. In 2018, Weissbart and colleagues found changes in brain activity by functional magnetic resonance imaging after 6 weeks of neuromodulation [10]. Neuromodulation was found to change brain activity and spinal cord reflexes leading to improvement in detrusor overactivity, bladder filling sensation, urge, and micturition timing [11, 12]. Different techniques of neuromodulation (sacral neuromodulation, transcutaneous tibial neuromodulation, percutaneous tibial neuromodulation, and pudendal anogenital neuromodulation) seem to show similar effects although the stimulation occurs at different sites in the body [12]. This prospective clinical interventional study was conducted to evaluate the effectiveness of biofeedback-assisted PFMT and electrostimulation versus two different methods of application of the peripheral neuromodulation technique: posterior tibial neuromodulation and anogenital neuromodulation in the treatment of MUI among women.

## Methods

**Fig. 1:**
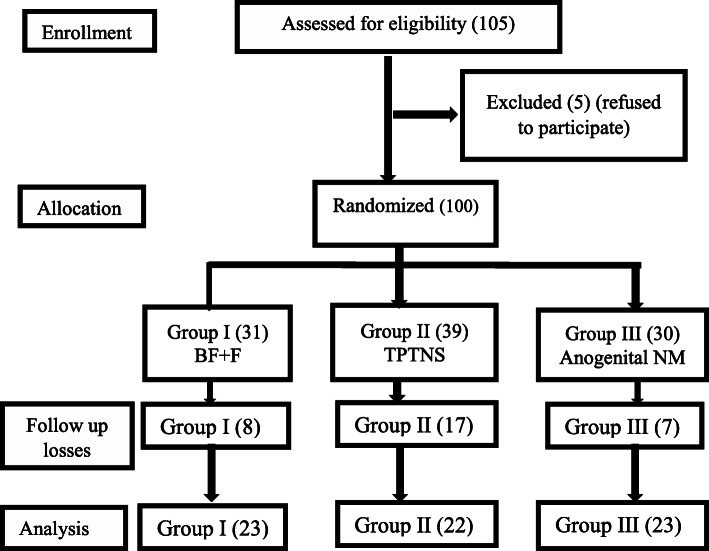
Flow chart of the participants. BF, biofeedback with electrical stimulation; F, faradic stimulation, TPTNM, transcutaneous posterior tibial nerve stimulation; NM, neuromodulation

This randomized clinical trial study was held over the course of 18 months. It included non-virgin female patients (married, widows, or divorced) with MUI (Fig. 1). Diagnosis of MUI was reached by history taking and calculating the score of the QUID questionnaire for both stress and urge incontinence. The study was explained to the participants and written informed consent was given by each participant. Patients with idiopathic MUI, who had symptoms of stress and urge urinary incontinence and whose ages were above 18 years old were included in the study. Exclusion criteria included patients who had previous anorectal, genitourinary, and gynecological surgeries, traumatic perineal injury, history of radiotherapy, patients consuming drugs that affect lower urinary tract function, any neurological conditions that affect sphincteric function, and patients with urinary tract infection or vaginitis. Patients with implanted cardiac pacemaker and defibrillator and patients with uncompensated heart disease or uncontrolled hypertension were also excluded. Exclusion criteria were fulfilled by history taking, clinical examination, and urine analysis.

Patients were randomly allocated into three groups:

- **Group I** received biofeedback-assisted PFMT and faradic electrostimulation.
- **Group II** received neuromodulation in the form of bilateral transcutaneous posterior tibial nerve stimulation.
- **Group III** received neuromodulation in the form of anogenital electrical stimulation.

**Fig. 2:**
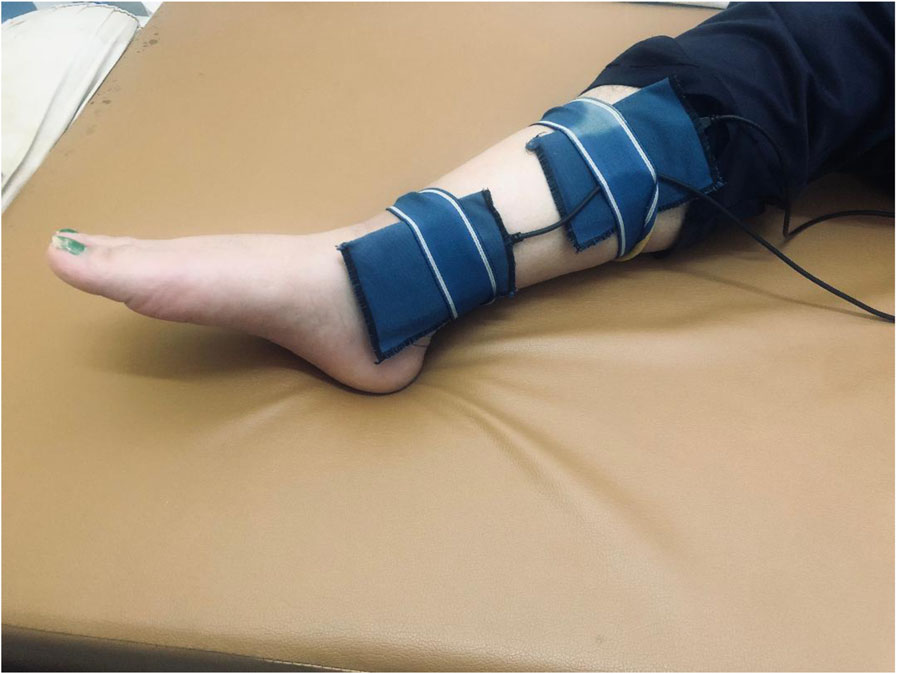
Posterior tibial neuromodulation

**Fig. 3:**
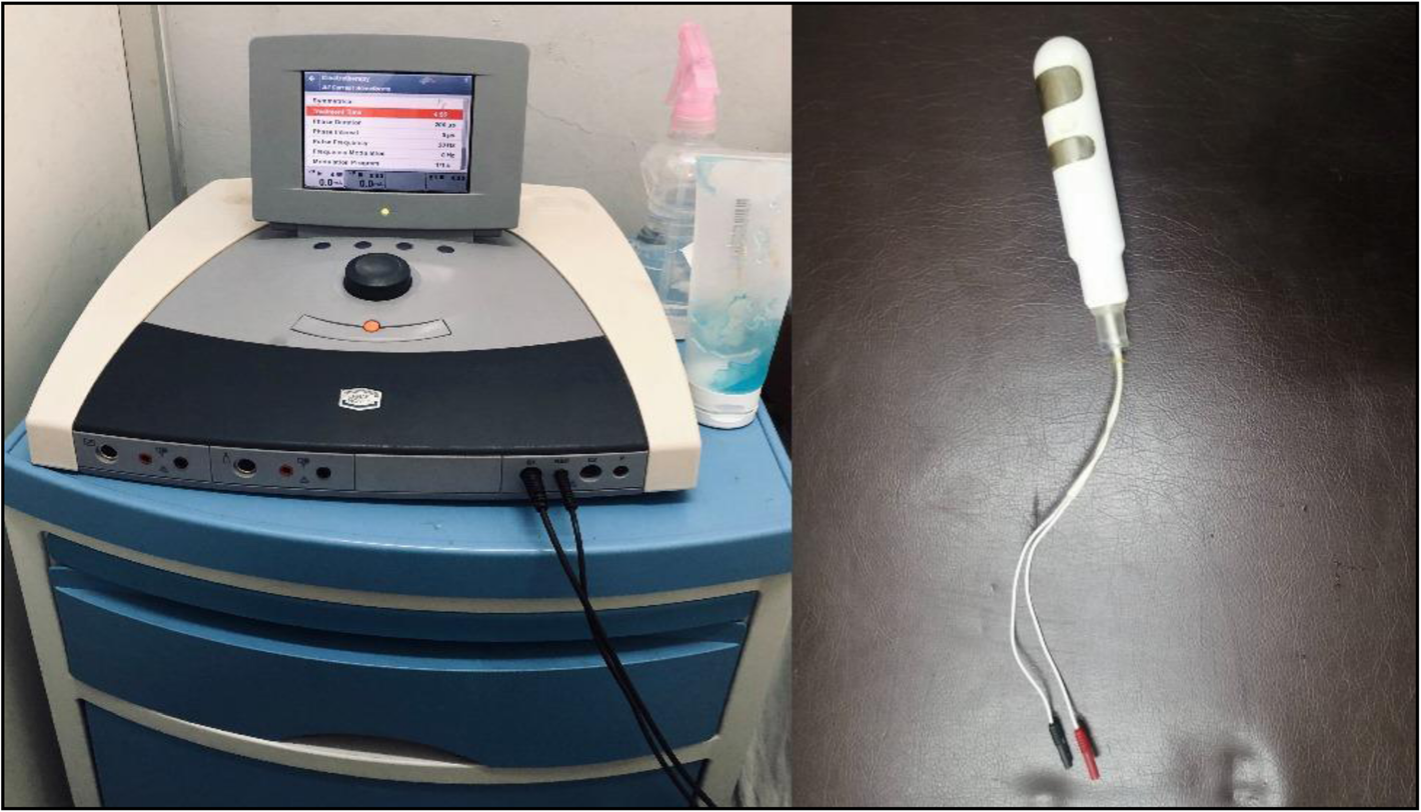
The biofeedback and electrostimulation device with the transvaginal electrical stimulating probe

All participants were subjected to:

1. History taking that included demographic data and history of the present condition concerning symptoms of urinary incontinence, duration of symptoms, and parity.
2. Baseline assessment questionnaires were applied and filled out by all patients to identify the severity of the problem and its impact on patients’ life:
  Questionnaire of female urinary incontinence diagnosis (QUID): It ranges from zero to 15 in stress and urge domain separately, stress scores ≥ 4 for stress urinary incontinence, and urge scores ≥ 6 for urge urinary incontinence are validated for diagnosis of the type of urinary incontinence [3].
  Australian Pelvic Floor Questionnaire (PFQ): It ranges from 0 to 40 according to the degree of pelvic floor dysfunction in urinary, bowel, prolapse, and sexual domains [13].
  International Consultation on Incontinence Questionnaire-Urinary Incontinence-Short Form (ICIQ-UI-SF) with a score ranging from 0 to 21. Score 0 means “no incontinence”, score ≥ 1 means “urinary incontinence”. The ICIQ-UI SF could be divided into the following four severity categories: slight (1–5), moderate (6–12), severe (13–18), and very severe (18–21) [14].
  Twenty-four-hour leaking episodes: Each patient was asked to record each time of urinary leakage through 24 hours [14].
3. Clinical examination of the perineum, rectum, and vagina was performed. Digital rectal examination and digital vaginal examination aimed to determine muscle power using the modified Oxford Muscle Grading System (MOS) [15].
4. Manometric pressure assessment was done for all patients to measure resting anal pressure, maximal anal and vaginal squeezing pressure. It was performed using the manometric biofeedback device (Myomed 632®, Enraf Nonius, Delft, Netherlands) according to the method described by Angelo [16].
5. Rehabilitation program: All patients were subjected to an individualized health education program, lifestyle modifications regarding weight loss, balanced diet, and home exercise program with Kegel’s exercise and avoidance of straining [17, 18].
  - Patients in group I were subjected to 12 sessions (three times weekly) of:
    Surged triangular faradic stimulation of the levator ani muscle using a transvaginal electrical stimulating probe for 15 min with 10-s holding time and 5-s interval. Pulse duration ranged from 0.1 to 1 ms (Fig. 3) [19]. The transvaginal electrical stimulating probe was sterilized after each use by washing with soap and water, then soaking in a cold water-based disinfectant such as Cidex 1% glutaraldehyde solution for 30 min followed by a water rinse to disperse the disinfecting agent.
    Pressure biofeedback-assisted PFMT was done using a vaginal pressure sensor covered with a sterile latex glove, inserted in the vagina 4 cm deep, and adjusting the pressure to 0 level before starting the exercise. Strengthening exercise was performed by asking the patient to maximally contract her PFM for 5 s followed by relaxation for 10 s for 10 repetitions. Then, a resting period for 3–4 min before starting the endurance exercise. Endurance exercise was done by asking the patient to submaximally contract her PFM (about 50% of maximal contraction pressure measured by the pressure manometry device) for 1 min or for the maximum period that she could afford if less than 1 min, followed by a resting period for 1 min. This exercise was repeated also 10 times. The strength of PFM was measured through the squeezing force in hectopascals (hPa), as well as the maximum duration of contraction was discussed with the patient each session to encourage her for better performance. Verbal encouragement response was given to the patient during performing the session. Visual and auditory clues were also provided to the patient as feedback to her PFM contraction [20, 21].
  - Patients in group II were subjected to 18 sessions (three times weekly) of bilateral TPTNS using (Myomed 632®, Enraf Nonius, Delft, Netherlands) machine, the active rubber surface was placed behind the medial malleolus and the reference electrode was placed 10 cm proximal (Fig. 2). Adjustment of the electric current was as follows: continuous TENS current, pulse duration 200 ms, frequency 20 Hz; each session lasts for 30 min. The current intensity was adjusted according to the tolerance of the patient or until the big toe curls into plantar flexion [22, 23].
  - Patients in group III were subjected to 18 sessions (three times weekly) of anogenital vaginal electrical stimulation using a transvaginal electrical stimulating probe and the same previous TENS current parameters (Fig. 3). The sessions were postponed during menstruation [24, 25].
6. Follow-up assessments at the end of the rehabilitation program were done in the form of subjective outcome measures (questionnaires) and objective measures (clinical examination, manometric pressure measurements). According to the findings of the outcome measures at the time of follow-up assessment, patients were categorized as having the following: [26]
  Complete improvement: It was defined as the improvement of all subjective and objective outcome measures.
  Partial improvement: Improvement of at least 50% of the subjective and objective outcome measures.
  No improvement: Neither improvement in the subjective nor the objective measures, or improvement in less than 50% of the subjective and objective outcome measures.
7. Statistical analysis: Data was introduced to the computer and analyzed using the IBM SPSS software package version 25.0. Qualitative data were described using numbers and percentages. Quantitative data were described using range (minimum and maximum), mean, standard deviation, and median. The distributions of quantitative variables were tested for normality using the Kolmogorov-Smirnov test. Non-parametric tests were used. A comparison between different groups regarding categorical variables was tested using the chi-square test. The Kruskal-Wallis test was used to compare the three studied groups. The Wilcoxon signed-rank test was used to compare the pre-intervention and post-intervention parameters in each group. Statistical significance was assigned to any *p* value ≤ 0.05 [27].

## Results

One hundred and five patients were recruited to participate in the current work, the flow of the recruited patients is demonstrated in Fig. 1. There was missing follow-up information for 32% of participants. Most of this loss of follow-up was due to the COVID-19 pandemic and the lockdown period. Other causes included: long duration of treatment, lack of motivation, traveling, and one patient had uterine carcinoma. Sixty-eight patients completed the study. The three studied groups were homogenous regarding demographic and clinical data. The mean age in years was (46.2±11.2, 45.6 ± 1.2, 50.3 ± 11.3) in groups I, II, and III respectively with an insignificant *p* value = 0.185. Body mass index ranged from 23 to 49 kg/m^2^, the mean value was (33.14 ± 4.8, 35.5 ± 6.11, 35.1 ± 4.7) in groups I, II, and III. No statistically significant *p* value was recorded (*p* = 0.297). Most of the patients in the three studied groups in the present work were multipara; the median value of parity was 4.

Values of assessment questionnaires before the intervention showed no statistically significant difference between the studied groups. Non-significant *p* values were reported between the three studied groups regarding QUID (*p* = 0.337), Australian PFQ (*p* = 0.965), and ICIQ-UI-SF (*p* = 0.564). Regarding 24-h leaking episodes, the median value was 4 and the *p* value = 0.983.

Most of the patients in the three studied groups before the intervention fell in the section of grades 3 and 4 of the MOS in the rectal and vaginal examination as shown in Table 3 without significant difference. Manometric pressure parameters before the intervention are assessed and tabulated in (Table 1).

**Table 1:** Comparison of the manometric pressure assessment in the three groups before the intervention

Manometric pressure before intervention	Group I (***n*** = 23)	Group II (***n*** = 22)	Group III (***n*** = 23)	Test of sig.	***p***
**Anal resting pressure (hPa)**					
Min.–max.	20–135	13–170	15–140	*H* = 2.9	0.223
Mean ± SD	61.2 ± 30.3	72 ± 40.4	81.3 ± 38.8		
Median	63	66.5	65		
**Anal max. squeezing pressure (hPa)**					
Min–max.	15–140	4–120	3–75	*H* = 1.2	0.530
Mean ± SD	54 ± 30.7	51.6 ± 40.2	41.7 ± 18		
Median	50	37.5	43		
**Vaginal max. squeezing pressure (hPa)**					
Min.–max.	10–80	5–45	11–80	*H* = 1.7	0.408
Mean ± SD	34 ± 16.7	27.7 ± 12.2	29.5 ± 15.6		
Median	30	25.5	25		

*Min*. minimum, *Max*. maximum, *n* number of patients, *hPa* for hectopascal

*H* and *p* values for Kruskal-Wallis test for comparing the three groups

*For statistical significance at *p* ≤ 0.05

There was a statistically significant difference in the assessment questionnaires before and after intervention in each of the studied groups (Table 2). The change in the MOS grading per rectal and vaginal examination showed a statistically significant difference before and after the intervention in the three studied groups (Table 3). Manometric pressure examination showed a significant increase in the resting anal pressure, maximal squeezing anal pressure, and maximal vaginal pressure in group I and group III. Group II did not show a statistical difference before and after the intervention regarding all manometric parameters. These findings were assessed and tabulated in Table 4.

**Table 2:** Comparison of the assessed questionnaires before and after the intervention among the three studied groups

Questionnaire	Group I		Test of sig.	***p***	Group II		Test of sig.	***p***	Group III		Test of sig.	***p***
	Before intervention	After intervention			Before intervention	After intervention			Before intervention	After intervention		
**QUID (SUI)**												
Min.–Max.	4–15	0–15	*Z* = − 3.5	≤ 0.0001*	4–15	0–15	*Z* = − 3.6	≤ 0.0001*	5–15	0–15	*Z* = − 3.74	≤ 0.0001*
Mean ± SD	9.7 ± 3.87	5.39 ± 4.4			9 ± 2.67	4.59 ± 4.2			10.3 ± 3	5.2 ± 5		
Median	9	4			9	3			10	3		
Percentage of improvement 43.6%					52.4%				51.3%			
**QUID (UUI)**												
Min.–Max.	6–15	0-15	*Z* = − 3.7	≤ 0.0001*	6–15	0–15	*Z* = − 3.7	≤ 0.0001*	7–15	0–15	*Z* = − 3.64	≤ 0.0001*
Mean ± SD	11.1 ± 2.96	5.7 ± 4.1			12 ± 2.81	5.9 ± 4.5			12.4 ± 2.38	6.13 ± 4.98		
Median	12	4			12	3.5			12	3		
Percentage of improvement 47.8%					52.2%				49%			
**QUID (Total)**												
Min.–Max.	10–30	2–27	*Z* = − 3.7	≤ 0.0001*	15–30	0–30	*Z* = − 3.8	≤ 0.0001*	17–30	2–30	*Z* = − 3.7	≤ 0.0001*
Mean ± SD	20.9 ±5.6	11.1 ± 8			21 ± 4.1	10.5 ± 8.5			22.7 ± 4	11.3 ± 9.7		
Median	22	8			19.5	6			24	6		
Percentage of improvement 45.7%					52%				50.7%			
**Global PFQ**												
Min.–Max.	4–21.7	0.67-23.73	*Z* = − 3.4	0.001*	4.1–22.8	1-23.86	*Z* = 3.8	≤ 0.0001*	4.5–23.2	0.52–26.1	*Z* = − 3.28	0.001*
Mean ± SD	14.5± 5.3	11.3 ±6.5			14.2 ± 5.7	11 ± 6.3			14.1 ± 6	10.8 ± 7.6		
Median	15.7	11.9			15	11.2			13.8	10.6		
Percentage of improvement 28.1%					24.6%				28%			
**ICIQ-UI-SF**												
Min.–Max.	7-23	0-22	*Z* = − 3.6	≤ 0.0001*	9–21	0–19	*Z* = 4.18	≤ 0.0001*	9–21	2–21	*Z* = − 3.88	≤ 0.0001*
Mean ± SD	9.7 ± 3.87	8.8±6.54			15.2 ± 3.6	8.8 ± 5.3			14 ± 3.6	7.8 ± 5.7		
Median	9	6			14.5	9			13	6		
Percentage of improvement41.2*%*					43.4%				46.5%			
**24-h leaking episodes**												
Min.–Max.	2–8	0–7	*Z* = − 3.6	≤ 0.0001*	3–7	0–7	*Z* = − 3.4	0.001*	2–8	0–6	*Z* = − 3.86	≤ 0.0001*
Mean ± SD	4.3 ± 1.7	1.9 ± 2.1			4.18 ± 1.2	2 ± 1.9			4.3 ± 1.6	1.8 ± 1.97		
Median	4	1			4	1			4	1		
Percentage of improvement 54.2%					58.3%				66.4%			

*QUID* Questionnaire for female Urinary Incontinence Diagnosis, *SUI* stress urinary incontinence, *UUI* urge urinary incontinence, *PFQ* pelvic floor questionnaire, *ICIQ-UI-SF* International Consultation on Incontinence Questionnaire-Urinary Incontinence-Short Form, *Min.* minimum, *Max.* maximum, *n* number of patients, *SD* for standard deviation

*Z* and *p* values for the Wilcoxon signed-rank test for comparing two related samples

*Statistically significant at *p* ≤ 0.05

**Table 3:** Comparison of the Modified Oxford Muscle Grading System before and after the intervention per rectum and per vaginal examination among the three studied groups

MOS	Group I				Test of sig.	p	Group II				Test of sig.	p	Group III				Test of sig.	p
	Before intervention		After intervention				Before intervention		After intervention				Before intervention		After intervention			
	n	%	n	%			(n)	%										
							n	%	n	%			n	%	n	%		
**Per rectum**																		
**1**	0	0%	0	0%	Z = − 2.8	0.004*	0	0%	0	0%	Z = − 2.97	0.003*	1	4.3%	0	0%	Z = − 3.36	0.001*
**2**	0	0%	0	0%			1	4.5%	0	0%			0	0%	0	0%		
**3**	9	39.1%	4	17.4%			15	68.2%	6	27.3%			15	65.2%	7	30.4%		
**4**	13	56.5%	13	56.5%			6	27.3%	15	68.2%			6	26.1%	13	56.5%		
**5**	1	4.3%	6	26.1%			0	0%	1	4.5%			1	4.3%	3	13.0%		
Percentage of improvement 8.7%							10.9%						11.3%					
**Per vagina**																		
**1**	0	0%	0	0%	Z = − 3.2	0.001*	0	0%	0	0%	Z = − 3.16	0.002*	1	4.3%	0	0%	Z = − 3.36	0.001*
**2**	3	13%	1	4.3%			1	4.5%	1	4.5%			0	0%	0	0%		
**3**	13	56.5%	6	26.1%			15	68.2%	6	27.3%			18	78.3%	8	34.8%		
**4**	7	30.4%	12	52.2%			6	27.3%	14	63.6%			4	17.4%	12	52.2%		
**5**	0	0%	4	14.4%			0	0%	1	4.5%			0	0%	3	13.0%		
Percentage of improvement 13%							9%						14%					

*MOS* Modified Oxford Muscle Grading System, *n* number of patients, *%* for percentage

*Z* and *p* values for the Wilcoxon signed-rank test for comparison before and after intervention in the same group

*Statistically significant at *p* ≤ 0.05

**Table 4:** Comparison of the assessed manometric parameters before and after the intervention among the three studied groups

Manometric pressure	Group I				Group II				Group III			
	Before intervention	After intervention	Test of sig.	***p***	Before intervention	After intervention	Test of sig.	***p***	Before intervention	After intervention	Test of sig.	***p***
**Anal resting pressure (hPa)**												
Min.–Max.	20–135	20–130	*Z* = − 3.1	0.002*	13–170	20–140	*Z* = − 1	0.312	150–140	15–150	*Z* = − 3.08	0.002*
Mean ± SD	61.2 ± 30.3	72.4 ± 28.3			71.9 ± 40.45	77 ± 33.8			81.2 ± 38.8	88.6 ± 42.5		
Median	63	79			66.5	73			65	85		
Percentage of improvement	29.3%				25.8%				9.5%			
**Anal max. squeezing pressure (hPa)**												
Min.–Max.	15–140	13–156	*Z* = − 3.5	≤ 0.0001*	4–120	9–140	*Z* = − 1.5	0.130	3–75	9–90	*Z* = − 3.35	0.001*
Mean ± SD	54 ± 30.6	65.3 ± 35.4			51.6 ± 40.2	55.1 ± 39.5			41.7 ± 18	50.5 ± 21.1		
Median	50	63			37.5	42.5			43	60		
Percentage of improvement	23.8%				31.3%				29.5%			
**Vaginal max. squeezing pressure (hPa)**												
Min.–Max.	10–80	20–71	*Z* = − 2.8	0.004*	5–45	5–60	*Z* = − 1.1	0.269	11–80	15–90	*Z* = − 2.7	0.006*
Mean ± SD	34 ± 16.7	43 ± 13.5			27.7 ± 12.2	29.6 ± 14.4			29.5 ± 15.6	34 ± 16.3		
Median	30	42			25.5	28			25	30		
Percentage of improvement	47.8%				12.6%				21.5%			

*Min*. minimum, *Max*. maximum, *SD* standard deviation, *hPa* hectopascal

*Z* and *p* values for the Wilcoxon signed-rank test for comparison before and after intervention in the same group

*Statistically significant at *p* ≤ 0.05

### Comparisons between the three studied groups after the intervention

No statistically significant differences were detected between the three studied groups regarding assessment questionnaires, clinical examination, and manometric pressure assessment after the intervention.

Anal resting pressure and anal maximal squeezing pressure didn’t show statistically significant differences between the three studied groups, while maximal vaginal squeezing pressure showed a statistically significant difference between the three groups. The Mann-Whitney test was done between each 2 groups to detect the contributor of this difference. It revealed that group I showed a higher increase in maximal vaginal pressure rather than groups II and III (p I–II = 0.002, p I–III= 0.015, p II–III = 0.564) (Table 5).

**Table 5:** Comparison of the pressure manometric assessment in the three groups after the intervention

Pressure manometry after intervention	Group I (***n*** = 23)	Group II (***n*** = 22)	Group III (***n*** = 23)	Test of sig.	***p***
**Anal resting pressure (hPa)**					
Min.–Max.	20–130	20–140	15–150	*H* = 1.5	0.451
Mean ± SD	72.4 ± 28.3	77 ± 33.8	88.6 ± 42.5		
Median	79	73	85		
**Anal max. squeezing pressure (hPa)**					
Min.–Max.	13–156	9–140	9–90	*H* = 1.86	0.393
Mean ± SD	65.3 ± 35.4	55.1 ± 39.5	50.5 ± 21.1		
Median	63	42.5	60		
**Vaginal max. squeezing pressure (hPa)**					
Min.–Max.	20–71	5–60	15–90	*H* = 10.9	0.004*
Mean ± SD	43 ± 13.5	29.6 ± 14.4	34 ± 16.3		
Median	42	28	30		

*Min*. minimum, *Max.* maximum, *n* number of patients, *hPa* for hectopascal

*H* and *p* values for the Kruskal-Wallis test for comparing the three groups

*Statistically significant at *p* ≤ 0.05

**Table 6:** Frequencies of occurrence of improvement in the three studied groups after the intervention

Improvement	Group I (n = 23)		Group II (n = 22)		Group III (n = 23)		Test of sig.	p
	n	%	n	%	n	%		
**No improvement**	2	8.7%	6	27.3%	3	13%	X^2^ = 3.12	0.537
**Partial improvement**	12	52.2%	10	45.5%	11	47.8%		
**Complete improvement**	9	39.1%	6	27.2	9	39.1%		

*n* number of patients, *%* percentage

*X*^2^ and *p* values for the chi-square test for comparing the three groups

*Statistically significant at *p* ≤ 0.05

There was no statistically significant difference regarding the occurrence of improvement between the three studied groups (Table 6).

No significant correlations were noted between improvement following the intervention with age, parity, and BMI (Spearman’s correlation coefficient *p =* 0.293, 0.759, 0.604 respectively).

## Discussion

The current work was the first that compared biofeedback-assisted PFMT versus TPTNS and anogenital neuromodulation in MUI female patients. The three methods showed a statistically significant difference between pre- and post-intervention assessment; however, none of the three methods was inferior to the others. This could guide the rehabilitation physicians to choose freely between the three methods according to economic resources, safety and infection control issues as well as the invasiveness of the treatment method concerning the patients’ preferences.

In group I, 39.1% experienced complete improvement, 52.2% experienced partial improvement and 8.7% did not show any sign of improvement. In group II, 27.3% of patients showed complete improvement, 45.4% of patients showed partial improvement and 27.3% had no improvement. In group III, 39.1% of patients showed complete improvement, 47.8% of patients showed partial improvement, and only 13% of patients had no improvement.

Richmond and his colleagues conducted a study in 2016 on 16 women with MUI and 31 women with SUI to study the effect of supervised pelvic floor biofeedback and electrical stimulation. Participants received one session per week for 4 to 8 weeks. After treatment, MUI patients showed a significant increase in levator ani strength in comparison to patients with SUI. Both groups showed subjective improvement after treatment and a tendency to recommend this method of treatment to other patients [25]. The results of Richmond’s study supported the use of pelvic floor biofeedback and electrical stimulation for women with MUI, as they demonstrated improvement in distress urinary incontinence symptoms after treatment more than women with SUI [25].

Previous studies discussed the efficacy of biofeedback-assisted PFMT on different types of UI. A Cochrane systematic review (2011) included twenty-four trials that reported that UI patients who received biofeedback-assisted PFMT were less likely to report a failure of the treatment, this may be due to the close contact between the patient and the health professional. The outcome measures in the previous studies included QOL assessment questionnaires, subjective improvement, leakage episodes, measurement of pelvic floor muscle function [6]. The review revealed that biofeedback-assisted PFMT was superior to standard PFMT [6]. This was congruent with Imamura and colleagues in his systematic review in 2010 when he studied the non-surgical treatment of SUI [30]. Pressure manometry assessment in the current study improved after the intervention in the groups that had no statistically significant difference between the three groups regarding resting anal pressure, and maximal anal pressure, while vaginal squeezing pressure showed more improvement following the intervention in group I [28].

The improvement in the manometric pressure assessment parameters in groups I and III following the intervention could be due to the correct performance of PFMT. Application of the transvaginal electrical stimulating probe plays a role in teaching patients how to feel and contract the pelvic floor muscles in a correct way. Posterior tibial neuromodulation lacks direct contact with the perineal area, this could be the cause of the absence of a statistically significant improvement in the vaginal maximal squeezing pressure after the intervention in group II.

Comparison of the vaginal maximal squeezing pressure between the three studied groups following the intervention showed more improvement in group I. This could be due to the direct long-time contact between patients and physician during the biofeedback sessions, which lead to more encouragement to do the exercise properly. Unlike a study conducted in 2003 by Kienle et al. to compare biofeedback and electrostimulation in anal insufficiency. Kienle concluded that biofeedback training is probably superior to electrostimulation in the conservative treatment of anal sphincter insufficiency, the improvement was dependent on anal resting pressure and maximal anal squeezing pressure in the biofeedback group versus improvement of the resting anal pressure only in the electrostimulation group [18].

The literature that described changes of manometry parameters after biofeedback training and electrostimulation is generally inconsistent.

The results of the current work support findings from a study on 15 patients above 65 years old with urinary and fecal incontinence exposed to 12 sessions of TPTNS. They showed improvement in 87% of patients compared to the placebo group. Also, the change of ICIQ-SF and 24-h leaking episodes improvement are similar in both studies [29]. Transcutaneous posterior tibial nerve stimulation was studied also on 70 multiple sclerosis patients with overactive bladder. Patients received daily (20 min) sessions for 3 months. It showed 82.6% improvement after 30 days and 83.3% after 90 days regarding urgency and frequency reported by bladder diary and symptom score as a primary outcome measure [30].

Most of the previous studies of PTNM were carried out on patients with overactive bladder. Transcutaneous posterior tibial nerve stimulation was less likely used than percutaneous PTNM. A systematic review supports evidence for the effectiveness of PTNM on urinary symptoms, pain, and QoL measures of OAB and fecal incontinence [30]. Peters and his colleagues (2009) compared PTNM with tolterodine (pharmacological treatment) in treatment of overactive bladder, He concluded that PTNM may be considered a clinically significant alternative of medical treatment [31]. Abulseoud and his team conducted a study in 2017 to compare the effect of TPTNM alone versus combination with low dose trospium chloride in female patients with OAB, they concluded that combined therapy was more effective than TPTNM alone [32].

The results in group III in the current work go in parallel with the results of Barroso and his colleagues, who conducted a research on transvaginal electrical stimulation on 36 women (24 patients and 12 controls) who suffered from different types of UI. They received home-based, 20-min sessions, twice daily for 12 weeks. Follow-up after 6 months revealed that the treatment group had a significant decrease in the number of leaking episodes, number of voids, and number of episodes of urgency. Barroso et al. recommended anogenital neuromodulation as a safe effective method to manage UI in females [33]. Regarding the exact number of treatment sessions with anogenital neuromodulation in the literature, it is not well established. Primus and Kramer found that certain patients with neurogenic and non-neurogenic incontinence didn’t improve until after receiving five treatments, and they suggested that at least ten sessions should be provided [34].

Previous research projects were conducted to study the effect of vaginal neuromodulation on SUI, most of them showed superiority in comparison to placebo. While others concluded that this method of treatment was effective only in treating UUI [35].

A recent study was done on 106 women with UUI and MUI to study the effect of the addition of vaginal neuromodulation to TPTNS in comparison to TPTNS alone. Both methods were effective in improving symptoms of incontinence and QOL. And the double therapy group showed a dropout rate three times more than the TPTNS group, which reflects the better acceptance of the patients to the less exposing easy method of TPTNS [36]. This was different from the current work, as a higher dropout rate was noticed with group II. This may be due to the social culture that recommends the application of the treatment directly to the diseased area.

The mechanism of action of biofeedback-assisted PFM training depends on its direct strengthening effect on the pelvic floor muscles, in addition to improving the contraction techniques by showing the patients the actual activity of their pelvic floor muscles in real-time. Therefore, it has the indirect effects of motivating patients and increasing their adherence to the exercise program [37]. Biofeedback-assisted PFMT can increase urethral pressure, leading to the inhibition of the sacral preganglionic innervation to the bladder through the guarding reflex. Pelvic floor muscle contraction can also stimulate the sympathetic nerve fibers of the internal urethral sphincter, thereby causing a decrease in detrusor muscle pressure. Pelvic floor muscle contraction can also stimulate the sympathetic nerve fibers of the internal urethral sphincter, causing a decrease in detrusor muscle pressure [38]. When faradic stimulation is added to biofeedback-assisted PFM training, the number of slow twitching fibers increases, replacing the fast twitching fibers, resulting in a more durable and rigorous sphincter muscular contraction. Patients can feel the contraction of distinct muscle groups and experience the feeling essential for normal activity of the PFMs during an electrical stimulation session, which leads to better continence [39].

Neuromodulation works by stimulating peripheral somatic afferent nerves, such as the posterior tibial, pudendal, or sacral nerves. Stimulation of the peripheral afferent nerve blocks competing abnormal visceral afferent signals from the bladder and prevents reflex bladder hyperactivity or retention [40]. Neuromodulation also affects spinal afferents, preganglionic sympathetic, and parasympathetic efferents [41]. The impulses move to the brain stem and cerebral centers for bladder control via spinal pathways, with the final impact of modulating reflex pathways [42, 43]. The exact mechanism of neuromodulation is still unclear.

This study has some limitations. The diagnosis of MUI depending on patients' complaints was made using assessment questionnaires by interview, and not by standard urodynamic diagnosis. The current study needs to be continued to confirm or refute the long-term effects of results. The conclusions of the current study are limited by a relatively small sample size of patients, which does not permit adequately powered comparisons between subgroups; however, these results can be used to power future comparative studies.

## Conclusions

According to the current findings, all studied MUI patients showed significant subjective and objective improvement following the rehabilitation program. Biofeedback-assisted PFM training is as effective as posterior tibial neuromodulation and anogenital neuromodulation in the treatment of MUI among women**.**

## Data Availability

All are available with the corresponding author.

## Abbreviations

MUI: Mixed urinary incontinence
QOL: Quality of life
PFMT: Pelvic floor muscle training
TPTNS: Transcutaneous posterior tibial nerve stimulation
QUID: Questionnaire for female Urinary Incontinence Diagnosis
PFQ: Pelvic floor questionnaire
ICIQ-UI-SF: International Consultation on Incontinence Questionnaire-Urinary Incontinence-Short Form
UI: Urinary incontinence
BF: Biofeedback
TENS: Transcutaneous electrical nerve stimulation
BMI: Body mass index
EAU: European Association of Urology
